# Effects of DRG/DIP payment reform on hospital pharmacy administration and pharmaceutical services in China: a multicenter cross-sectional study

**DOI:** 10.3389/fpubh.2025.1585279

**Published:** 2025-07-11

**Authors:** Xuanxuan Wang, Yun Tao, Suyu Gao, Jiajia Feng, Anqi Huang, Likai Lin, Hong Cheng

**Affiliations:** ^1^Department of Pharmacy, Zhongnan Hospital of Wuhan University, Wuhan, China; ^2^Hospital Management Institute of Wuhan University, Zhongnan Hospital of Wuhan University, Wuhan, China

**Keywords:** diagnosis-related groups, diagnosis-intervention packet, healthcare payment reform, pharmacy administration, pharmaceutical services, multicenter study

## Abstract

**Background:**

In China, a government-led policy introduced in 2019 and 2020 aims to reduce medical costs through a national medical care payment system based on Diagnostic-Related Groups (DRG) and Diagnosis-Intervention Packet (DIP). Hospital pharmacists play a crucial role in the implementation of this policy by enhancing the rational use of medicines and delivering pharmaceutical services. The purpose of this study is to assess the current state of hospital pharmacy administration and pharmaceutical services, while examining the effects of the DRG/DIP policy on these aspects.

**Methods:**

This multicenter cross-sectional study utilized a questionnaire survey to collect data. The questionnaire consisted of four main parts: participants' demographics, hospital and DRG/DIP payment information, hospital pharmacists' involvement in pharmacy administration and pharmaceutical services, and barriers and suggestions encountered in their work. The questionnaire was distributed to pharmacy department heads in hospitals across mainland China through convenience sampling, between September 2022 and December 2022. Multivariate logistic regression analysis was performed to identify factors associated with hospital pharmacy administration and pharmaceutical services.

**Results:**

A total of 655 pharmacists from 655 hospitals participated in the questionnaire survey. Pharmacists in DRG/DIP implemented hospitals were more involved in both pharmacy administration and pharmaceutical services compared to those in non-DRG/DIP implemented hospitals. The DRG/DIP reform was associated with improved hospital pharmacy administration (OR = 1.87, 95% CI 1.26–2.77, *p* = 0.002). Additionally, favorable outcomes in pharmaceutical services were associated with the DRG/DIP reform (OR = 1.79, 95% CI 1.07–3.00, *p* = 0.027) and enhanced pharmacy administration (OR = 28.10, 95% CI 17.61–44.85, *p* < 0.001).

**Conclusions:**

To effectively adapt the healthcare payment reform, it is suggested that the pharmacy department should adopt DRG/DIP as a strategic focus, continuously enhance pharmaceutical services capabilities and pharmacy administration systems, and achieve value optimization within the context of healthcare payment reform.

## 1 Introduction

The increasing demand for medical resources has led to a rapid increase in medical expenses in China (1, 2). From 2015 to 2022, China's total health expenditure increased from 40,974.64 to 85,327.49 billion yuan, with an annual growth rate of 11.05% (3). China's real per capita health expenditure increased from 2,962.20 to 6,044.10 yuan during the same period, with an annual growth rate of 10.72% (3), which outpaced the annual growth rate of per capita gross domestic product (GDP) at 8.03% (4). Recognizing the urgent need to control these rising medical costs, many low- and middle-income countries have actively adopted Diagnosis-Related Group (DRG) payment system as a crucial cost-containment mechanism (5). In China, a new medical insurance payment system, known as the Diagnosis-Intervention Packet (DIP), was introduced to alleviate the financial burden of medication on hospitalized patients (6).

China's National Healthcare Security Administration launched DRG pilot programs in 30 designated regions in May 2019, and DIP pilots in 71 regions in October 2020 (7). In November 2021, China's National Healthcare Security Administration formulated a 3-year action plan for healthcare payment reform, proposing to achieve full DRG/DIP coverage for all eligible medical institutions by the end of 2025 (8). In 2021, all 101 pilot cities had entered the actual payment stage. By the end of 2022, 206 cities have achieved actual payment of DRG/DIP, and the proportion of medical insurance fund paid by DRG/DIP to the inpatient medical insurance fund expenditures reached 77% (9). Currently, as of 2024, DRG/DIP payment has been achieved to cover all overall planning regions, and the proportion of medical insurance fund expenditure paid by DRG/DIP accounts for more than 80% of inpatient medical insurance fund expenditures (10). DRG/DIP payment has achieved a significant leap from local pilot to national coverage, offering institutional support for the sustainable development of medical insurance funds. Studies conducted in China have indicated that DRG/DIP payment reform can effectively reduce average hospitalization costs (5, 6, 11, 12). Amid ongoing reforms in the medical insurance payment system, new requirements have emerged for hospital pharmacy administration and pharmaceutical services. Hospitals pharmacists are expected to play a key role in controlling medical costs by using DRG/DIP payment to optimize medical resource utilization while ensuring safety and efficacy (13).

Pharmaceutical services refers to pharmacists' contributions to patient care aimed at optimizing medication use and improving health outcomes (14). Over the years, the importance of pharmaceutical services has grown worldwide. Many countries have incorporated pharmaceutical services into their healthcare systems to improve clinical outcomes and reduce economic burdens (15–19). However, pharmaceutical services development in China lags behind that in Western countries. Hospital-based pharmaceutical services is still in early stage, with most hospitals yet to fully recognize its value in clinical practice (20). In response to healthcare payment reform, China's National Health Commission has issued a series of policies and regulations, including the *Opinions on accelerating the high-quality development of pharmaceutical services* and the *Opinions on strengthening pharmacy administration in medical institutions to promote rational drug use* (21, 22). These initiatives aim to shift the hospital pharmaceutical services model from a “drug-centered” approach to “patient-centered” one, and from focusing solely on drug supply to a model that ensures drug supply while emphasizing professional pharmaceutical services and active clinical participation. Hospital pharmacy administration and pharmaceutical services are now undergoing a critical period of transformation and advancement (23, 24).

Despite the growing implementation of DRG/DIP payment reform in China, domestic researches on hospital pharmacy administration and pharmaceutical services in DRG/DIP payment reform remains limited (25). To the best of our knowledge, no studies have systematically investigated the effects of DRG/DIP payment reform on hospital pharmacy administration and pharmaceutical services. This study aims to assess the current state of hospital pharmacy administration and pharmaceutical services in China, while exploring the effects of DRG/DIP payment reform on these aspects. Meanwhile, it seeks to investigate the barriers and propose corresponding suggestions for pharmacy work under the healthcare payment reform.

## 2 Materials and methods

### 2.1 Study design

This study is a multicenter cross-sectional study (26) based on a questionnaire survey, targeting hospital pharmacists across different regions in China. The study protocol has been approved by the Ethics Committee of Zhongnan Hospital of Wuhan University (Approval Number: 2022082K).

### 2.2 Setting

This analysis was initiated and conducted at the Department of Pharmacy of Zhongnan Hospital of Wuhan University, from September 28 to December 25, 2022. This study employed convenience sampling, covering 27 provinces (autonomous regions) and four directly administered municipalities.

### 2.3 Sample size

To determine the sample size for this study, we utilized the widely used online sample size calculator Raosoft (27, 28). The margin of error was set to 4%, and the confidence level at 95% (29, 30). Based on the official 2021 statistics of 1,030,935 medical institutions in China (31), the required sample size for this study was calculated to be 600.

### 2.4 Inclusion and exclusion criteria

The inclusion criteria required pharmacy professionals employed in hospitals across different regions in China. These pharmacy professionals completed the questionnaire on behalf of their hospitals, so only one pharmacy professional was asked to complete the questionnaire in each hospital. The questionnaire using screeners that automatically excluded any respondents who did not provide electronic informed consent or complete the questionnaire.

### 2.5 Questionnaire design

A team of clinical pharmacists developed the questionnaire following an extensive literature review (15, 20, 32–34) and a series of expert discussions. The Chinese version of the questionnaire was used throughout the study, and the Chinese version and the English version are available in Supplementary material S1. The contact information and email addresses were provided in the questionnaire instructions. Respondents can contact us at any time to clarify doubts during questionnaire completion. A pilot survey was conducted among 70 pharmacists in 4 hospitals to assess reliability and validity. For scale-based questions (Questions 5–10 in Section III), the reliability analysis showed a Cronbach's alpha of 0.923, indicating excellent internal consistency (35). The content validity was reviewed by three experts in the field (two from pharmacoepidemiology, and one from health policy). And Kaiser-Meyer-Olkin (KMO) and Bartlett's sphericity test revealed the KMO value was 0.866 (>0.8, *p* < 0.05), demonstrating excellent validity (23). Minor language adjustment was made when respondents indicated challenge of comprehension.

The questionnaire comprised 21 questions divided into four sections: (1) personal information: collects respondents' demographic data, consisting of five questions; (2) medical institution information: gathers details about the hospital where the respondents work, including four questions; (3) current status of pharmacy administration and pharmaceutical services in medical institutions: examines the overall implementation of pharmacy administration and pharmaceutical services under DRG/DIP payment reform. Respondents evaluated hospital pharmacy administration and pharmaceutical services using a 5-level Likert scale: 1 = “strongly disagree,” 2 = “disagree,” 3 = “neutral,” 4 = “agree,” and 5 = “strongly agree;” and (4) challenges and recommendations: contains two multiple-choice, seeking respondents' opinions and suggestions on policy improvements.

### 2.6 Variables

Sociodemographic data including age, gender, educational level, professional title, and work experience were collected. Hospital information such as grade, category, bed capacity, geographic location, medical insurance payment methods, pharmacy administration, and pharmaceutical services provided were also concurrently gathered. The implementation effect of hospital pharmacy administration was evaluated by respondents based on pharmacy management system, pharmaceutical care practice standards and the involvement of pharmacy department under DRG/DIP payment reform. Similarly, the implementation effect of hospital pharmaceutical services was assessed by respondents according to patient treatment, cost control and rational drug use. The options for the above six questions ranged from “strongly agree” to “strongly disagree,” with point values assigned from 5 to 1. Items scoring 4 or above on a 5-point Likert scale were considered to indicate good outcomes. The scores for pharmacy administration or pharmaceutical services of each hospital were calculated separately, falling within the range of 3–15. A score of ≥12 is considered indicative of a favorable level of pharmacy administration or pharmaceutical services.

### 2.7 Data collection

Between September 28 and December 25, 2022, to ensure that each hospital submitted only one response, the questionnaire was initially distributed exclusively to the heads of hospital pharmacy departments. These department heads subsequently designated a pharmacy professional to complete the questionnaire on behalf of their respective hospitals. The survey's progress was monitored biweekly until the deadline.

The questionnaires were distributed online via Wenjuanxing platform (https://www.wjx.cn/). Respondents were informed of the purpose and significance of the survey before participation. Participation was voluntary and anonymous, and all respondents provided electronic informed consent before completing the questionnaire.

### 2.8 Data analysis

Data analysis was conducted using Stata. Descriptive statistics were used to summarize demographic variables, using percentages or frequencies to demonstrate categorical variables. The Chi-square test was used to examine differences between groups. The multiple logistic regression analysis was performed to explore factors associated with good implementation effects of pharmacy administration and pharmaceutical services. The results were considered statistically significant when *p* < 0.05 and 95% confidence interval.

## Results

### 3.1 Characteristics of hospitals

**Figure 1:**
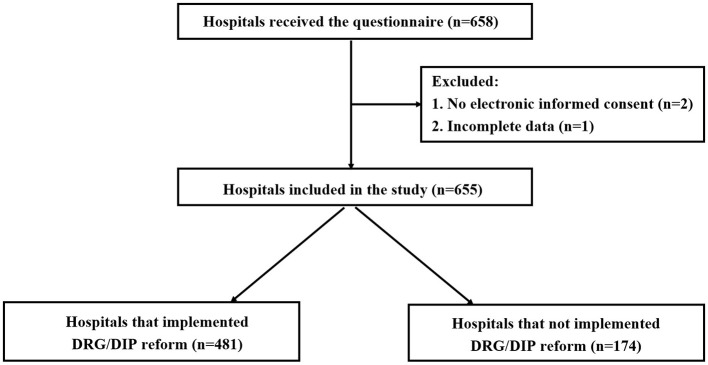
Flow chart of the enrolled hospitals.

A total of 658 questionnaires were distributed in this study, and 655 valid questionnaires were recovered, with a recovery rate of 99.5%. The flow chart of the enrolled hospitals was presented in Figure 1. 655 hospitals cover all provinces/autonomous regions/municipalities in China except Taiwan Province.

Table 1 presents the characteristics of hospitals. Among the 655 hospitals involved, 55.0% were tertiary hospitals, 88.9% were general hospitals, and 8.2% with ≥3,000 beds. In terms of geographical distribution based on economic division, the central region had the highest number of medical institutions, totaling 330 (50.4%), followed by the western region with 231 (35.3%), and the eastern region with lowest count of 94 (14.4%). Of the 655 medical institutions, 481 (73.4%) had implemented DRG/DIP payment reform, while 174 (26.6%) had not.

**Table 1:** Characteristics of hospitals.

**Variables**	***n* (%) *N* = 655**	**DRG/DIP implemented group, *n* (%) *N* = 481**	**Non-DRG/DIP implemented group, *n* (%) *N* = 174**	***p-*Value^a^**
**Hospital grade**				
Tertiary hospital	360 (55.0)	304 (63.2)	56 (32.2)	**<0.001**
Non-tertiary hospital	295 (45.0)	177 (36.8)	118 (67.8)	
**Hospital category**				
General hospital	582 (88.9)	426 (88.6)	156 (89.7)	0.70
Specialized hospital	73 (11.1)	55 (11.4)	18 (10.3)	
**Number of hospital beds**				
≥3,000	54 (8.2)	49 (10.2)	5 (2.9)	**0.003**
<3,000	601 (91.8)	432 (89.8)	169 (97.1)	
**Region**				
East	94 (14.4)	77 (16.0)	17 (9.8)	**0.012**
Middle	330 (50.4)	250 (52.0)	80 (46.0)	
West	231 (35.3)	154 (32.0)	77 (44.2)	

The bold values are statistically significant data, mainly for ease of viewing.

a Using Chi-square test.

Comparison between DRG/DIP implemented hospitals and non-DRG/DIP implemented hospitals revealed a higher proportion of tertiary hospitals and hospitals with ≥3,000 beds in the former group (*p* < 0.01). Additionally, variations in geographical distribution were noted (*p* = 0.012), although no statistical difference was observed in hospital category.

### 3.2 Characteristics of respondents

Characteristics of respondents are shown in Table 2. Among the 655 respondents, 245 were male (37.4%) and 410 were female (62.6%), for a male-to-female ratio of 1:1.55, The respondents were distributed across all age groups, with 76.5% being 50 years old or younger. 182 (27.8%) of the respondents held a master's or doctoral degree, and 313 (47.8%) had a senior professional title. The definitions and further details regarding the professional title categories are available in Supplementary material S1. Moreover, 478 (73.0%) had more than 10 years of experience in pharmacy work.

**Table 2:** Characteristics of respondents.

**Variables**	***n* (%) *N* = 655**	**DRG/DIP implemented group, *n* (%) *N* = 481**	**Non-DRG/DIP implemented group, *n* (%) *N* = 174**	***p-*Value^a^**
**Gender**				
Male	245 (37.4)	193 (40.1)	52 (29.9)	**0.02**
Female	410 (62.6)	288 (59.9)	122 (70.1)	
**Age**				
≤ 30	63 (9.6)	45 (9.4)	18 (10.3)	0.52
31–40	231 (35.3)	179 (37.2)	52 (29.9)	
41–50	207 (31.6)	144 (29.9)	63 (36.2)	
51–60	150 (22.9)	110 (22.9)	40 (23.0)	
>60	4 (0.6)	3 (0.6)	1 (0.6)	
**Education level**				
Doctor	43 (6.6)	36 (7.5)	7 (4.0)	**<0.001**
Master	139 (21.2)	120 (24.9)	19 (10.9)	
Bachelor	401 (61.2)	295 (61.3)	106 (60.9)	
Others	72 (11.0)	30 (6.2)	42 (24.1)	
**Professional title**				
Senior	150 (22.9)	115 (23.9)	35 (20.1)	**0.001**
Deputy senior	163 (24.9)	121 (25.2)	42 (24.1)	
Intermediate	209 (31.9)	170 (35.3)	39 (22.4)	
Junior	96 (14.7)	59 (12.3)	37 (21.3)	
Others	37 (5.6)	16 (3.3)	21 (12.1)	
**Experience in pharmacy work/year**				
>30	127 (19.4)	96 (20.0)	31 (17.8)	0.34
21–30	159 (24.3)	114 (23.7)	45 (25.9)	
11–20	192 (29.3)	144 (29.9)	48 (27.6)	
6–10	122 (18.6)	94 (19.5)	28 (16.1)	
≤ 5	55 (8.4)	33 (6.9)	22 (12.6)	

The bold values are statistically significant data, mainly for ease of viewing.

a Using Chi-square test.

Comparing respondents from DRG/DIP implemented hospitals and non-DRG/DIP implemented hospitals, there were more males in the former group (*p* = 0.02). Furthermore, differences were observed in the distribution of professional titles (*p* = 0.001) and educational levels (*p* < 0.001) between the two groups, while no statistical differences were observed in age and years of pharmacy work.

### 3.3 Medical insurance payment methods in hospitals

It is important to note that multiple medical insurance payment methods may be implemented within the same hospital. Among the 655 hospitals surveyed, 333 (50.8%) have adopted DRG payment, while 336 (51.3%) have implemented DIP payment. Totally, 481 (73.4%) have implemented DRG/DIP payment. Regarding the roles of pharmacy departments in the DRG/DIP payment management system, most hospital pharmacy departments (459/481 95.4%) have well-defined, including communication with clinical departments, hospital formulary management, clinical pathway development, as well as medication monitoring and medication evaluation index setting.

### 3.4 Pharmacy administration and pharmaceutical services in hospitals

Table 3 presents the pharmacy administration and pharmaceutical services provided by the hospital pharmacy departments. The definitions and further details regarding the categories presented in Table 3 (pharmacy administration and pharmaceutical services), are available in Supplementary material S1. In terms of pharmacy administration, among the 655 hospitals surveyed, the majority (over 85%) provided prescription evaluation, antimicrobial stewardship, and adverse drug event management. Additionally, more than 65% of hospitals participated in off-label medication management, hospital formulary management and interpreting drug policy for physicians. However, less than 50% of hospitals offered comprehensive medicine-use evaluation and pharmacy administration related to medical insurance payment methods, including clinical pathway development (36.6%) and preferred pharmacologic regimen development (28.2%). Notably, DRG/DIP implemented hospitals tend to offer a more diverse range of pharmacy administration compared to non-DRG/DIP implemented hospitals, with statistically significant differences observed (*p* < 0.01).

**Table 3:** Pharmacy administration and pharmaceutical services in hospitals.

**Pharmacy administration/pharmaceutical services**	***n* (%) *N* = 655**	**DRG/DIP implemented group, *n* (%) *N* = 481**	**Non-DRG/DIP implemented group, *n* (%) *N* = 174**	***p-*Value^a^**
**Pharmacy administration**				
Prescription evaluation	628 (95.9)	473 (98.3)	155 (89.1)	**<0.001**
Antimicrobial stewardship	624 (95.3)	465 (96.7)	159 (91.4)	**0.005**
Adverse drug event management	587 (89.6)	443 (92.1)	144 (82.8)	**0.001**
Off-label medication management	523 (79.8)	403 (83.8)	120 (69.0)	**<0.001**
Hospital formulary management	474 (72.4)	366 (76.1)	108 (62.1)	**<0.001**
Interpreting drug policy for physicians	448 (68.4)	349 (72.6)	99 (56.9)	**<0.001**
Comprehensive medicine-use evaluation	323 (49.3)	264 (54.9)	59 (33.9)	**<0.001**
Clinical pathway development	240 (36.6)	196 (40.7)	44 (25.3)	**<0.001**
Preferred pharmacologic regimen development	185 (28.2)	169 (35.1)	16 (9.2)	**<0.001**
**Pharmaceutical services**				
Medication consultant	615 (93.9)	458 (95.2)	157 (90.2)	**0.02**
Providing lectures on rational drug use for medical staff or patients	523 (79.8)	397 (83.5)	126 (72.4)	**0.004**
Pharmaceutical ward round	498 (76.0)	401 (83.4)	97 (55.7)	**<0.001**
Pharmaceutical consults	485 (74.0)	388 (80.7)	97 (55.7)	**<0.001**
Unit dose dispensing system	474 (72.4)	382 (79.4)	92 (52.9)	**<0.001**
Prescription review	437 (66.7)	343 (71.3)	94 (54.0)	**<0.001**
Multi-disciplinary treatment	379 (57.9)	313 (65.1)	66 (37.9)	**<0.001**
Pharmaceutical clinic	371 (56.6)	294 (61.1)	77 (44.3)	**<0.001**
Medication therapy management	299 (45.6)	249 (51.8)	50 (28.7)	**<0.001**
Pharmacy intravenous admixture	295 (45.0)	231 (48.0)	64 (36.8)	**0.011**
Therapeutic drug monitoring	238 (36.3)	203 (42.2)	35 (20.1)	**<0.001**
Pharmacy practice in e-hospital	197 (30.1)	164 (34.1)	33 (19.0)	**<0.001**
Intelligent pharmacy	197 (30.1)	164 (34.1)	33 (19.0)	**<0.001**
Pharmacogenetic testing	183 (27.9)	164 (34.1)	19 (10.9)	**<0.001**
Hospital medication home delivery service	115 (17.5)	98 (20.4)	17 (9.8)	**0.002**

The bold values are statistically significant data, mainly for ease of viewing.

a Using Chi-square test.

As for pharmaceutical services, among the 655 hospitals surveyed, the majority (over 85%) provided medication consultant. More than 65% of hospitals provided lectures on rational drug use for medical staff or patients, pharmaceutical ward round, pharmaceutical consults, unit dose dispensing system and prescription review. Over 50% of hospitals offered multi-disciplinary treatment and pharmaceutical clinic. Additionally, some hospitals (>30%, <50%) offered medication therapy management, pharmacy intravenous admixture, therapeutic drug monitoring, pharmacy practice in e-hospital, and intelligent pharmacy. However, less than 30% of hospitals provided pharmacogenetic testing and hospital medication home delivery service. Notably, DRG/DIP implemented hospitals exhibit statistically significant differences compared to non-DRG/DIP implemented hospitals, showing a wider range of pharmaceutical services (*p* < 0.05).

### 3.5 Predictors of favorable pharmacy administration and pharmaceutical services

The assessment of implementation effects of hospital pharmacy administration and pharmaceutical services by respondents is shown in Supplementary Table S1. Most respondents (66.1%−89.5%) had a positive attitude (including strongly agree and agree) toward the status of hospital pharmacy administration and pharmaceutical services. Very few respondents (≤ 6.7%) had a negative attitude (including strongly disagree and disagree) toward the above issues. Among the 655 hospitals surveyed, 413 (63.1%) hospitals were evaluated as favorable pharmacy administration, and 420 (64.1%) were assessed as favorable pharmaceutical services.

As for multivariate logistic regression analysis, characteristics of hospitals and respondents were included to construct a regression equation. The Omnibus test (*p* < 0.0001, *p* < 0.0001) and the Hosmer–Lemesho test (*p* = 0.289, *p* = 0.879) indicated successful establishment and good fitting of the model for pharmacy administration and pharmaceutical services. DRG/DIP payment reform was found to be a facilitating factor to improved hospital pharmacy administration (OR = 1.87, 95% CI 1.26–2.77, *p* = 0.002; see Table 4). However, characteristics of respondents on hospital pharmacy administration was not statistically significant. The independent predictors of good pharmaceutical services were DRG/DIP payment reform (OR = 1.79, 95% CI 1.07–3.00, *p* = 0.027) and good pharmacy administration (OR = 28.10, 95% CI 17.61–44.85, *p* < 0.001; Table 5). Characteristics of respondents did not have a significant correlation, except for the professional title of “others (respondents not attain a professional title)”, which has significant association with pharmaceutical services (OR = 5.53, 95% CI 1.12–27.41, *p* = 0.04; see Table 5).

**Table 4:** Predictors of favorable pharmacy administration in hospitals.

**Variables**	**Pharmacy administration**		**OR (95% CI)**	***p-*Value^a^**
	**Unfavorable**, ***n*** **(%)** ***N*** = **242**	**Favorable**, ***n*** **(%)** ***N*** = **413**		
**Characteristics of hospitals**				
**Hospital grade**				
Tertiary hospital	109 (45.0%)	251 (60.8%)	1.33 (0.87, 2.06)	0.19
Non-tertiary hospital	133 (55.0%)	162 (39.2%)	Ref.	
**Hospital category**				
General hospital	210 (86.8%)	372 (90.1%)	1.60 (0.87, 2.72)	0.08
Specialized hospital	32 (13.2%)	41 (9.9%)	Ref.	
**Number of hospital beds**				
>3,000	10 (4.1%)	44 (10.7%)	1.48 (0.67, 3.27)	0.33
≤ 3,000	232 (95.9%)	369 (89.3%)	Ref.	
**Region**				
East	27 (11.2%)	65 (15.7%)	Ref.	
Middle	109 (45.0%)	220 (53.3%)	0.72 (0.41, 1.24)	0.41
West	106 (43.8%)	128 (31.0%)	0.55 (0.31, 0.96)	0.31
**Classification**				
DRG/DIP implemented group	157 (64.9%)	324 (78.5%)	1.87 (1.26, 2.77)	**0.002**
Non-DRG/DIP implemented group	85 (35.1)	89 (21.5%)	Ref.	
**Characteristics of respondents**				
**Gender**				
Male	80 (33.1%)	165 (40.0%)	1.29 (0.91, 1.84)	0.15
Female	162 (66.9%)	248 (60.0%)	Ref.	
**Age**				
≤ 30	23 (9.5%)	40 (9.7%)	Ref.	
31–40	96 (39.7%)	135 (32.7%)	1.10 (0.49, 2.46)	0.82
41–50	69 (28.5%)	138 (33.4%)	1.44 (0.55, 3.78)	0.46
51–60	53 (21.9%)	97 (23.5%)	1.18 (0.38, 3.66)	0.78
>60	1 (0.4%)	3 (0.7%)	1.33 (0.10, 18.48)	0.83
**Education level**				
Doctor	9 (3.7%)	34 (8.2%)	Ref.	
Master	36 (14.9%)	103 (24.9%)	1.13 (0.46, 2.81)	0.46
Bachelor	131 (54.1%)	235 (56.9%)	0.77 (0.31, 1.91)	0.31
Others	66 (27.2%)	41 (9.9%)	0.74 (0.24, 2.25)	0.49
**Professional title**				
Senior	39 (16.1%)	111 (26.9%)	Ref.	
Deputy senior	101 (41.7%)	101 (24.5%)	0.69 (0.39, 1.22)	0.39
Intermediate	92 (38.0%)	117 (28.3%)	0.52 (0.26, 1.06)	0.26
Junior	56 (23.1%)	56 (13.6%)	0.80 (0.33, 1.94)	0.33
Others	9 (3.7%)	28 (6.8%)	2.19 (0.61, 7.79)	0.61
**Experience in pharmacy work/year**				
>30	46 (19.0%)	81 (19.6%)	Ref.	
21–30	51 (21.1%)	108 (26.2%)	1.21 (0.59, 2.49)	0.60
11–20	84 (34.7%)	108 (26.2%)	1.11 (0.46, 2.66)	0.82
6–10	43 (17.8%)	79 (19.1%)	1.70 (0.61, 4.74)	0.31
≤ 5	18 (7.4%)	37 (9.0%)	1.29 (0.35, 4.73)	0.70

The bold values are statistically significant data, mainly for ease of viewing.

a Using multiple logistic regression analysis.

**Table 5:** Predictors of favorable pharmaceutical services in hospitals.

**Variables**	**Pharmaceutical services**		**OR (95% CI)**	***p-*Value^a^**
	**Unfavorable**, ***n*** **(%)** ***N*** = **235**	**Favorable**, ***n*** **(%)** ***N*** = **420**		
**Characteristics of hospitals**				
**Hospital grade**				
Tertiary hospital	112 (47.7%)	248 (59.0%)	1.03 (0.58, 1.84)	0.91
Non-tertiary hospital	123 (52.3%)	172 (41.0%)	Ref.	
**Hospital category**				
General hospital	204 (86.8%)	378 (90.0%)	1.35 (0.66, 2.76)	0.40
Specialized hospital	31 (13.2%)	42 (10.0%)	Ref.	
**Number of hospital beds**				
>3,000	16 (6.8%)	38 (9.0%)	0.42 (0.17, 1.01)	0.05
≤ 3,000	219 (93.2%)	382 (91.0%)	Ref.	
**Region**				
East	27 (11.5%)	65 (15.5%)	Ref.	
Middle	109 (46.4%)	220 (52.4%)	0.95 (0.47, 1.92)	0.90
West	99 (42.1%)	135 (32.1%)	0.91 (0.44, 1.88)	0.80
**Classification**				
DRG/DIP implemented group	152 (64.7%)	329 (78.3%)	1.79 (1.07, 3.00)	**0.027**
Non-DRG/DIP implemented group	83 (35.3%)	91 (21.7%)	Ref.	
**Characteristics of respondents**				
**Gender**				
Male	83 (35.3%)	162 (38.6%)	0.91 (0.57, 1.44)	0.67
Female	152 (64.7%)	258 (61.4%)	Ref.	
**Age**				
≤ 30	23 (9.8%)	40 (9.5%)	Ref.	
31–40	91 (38.7%)	140 (33.3%)	0.78 (0.26, 2.32)	0.66
41–50	71 (30.2%)	136 (32.4%)	0.97 (0.26, 3.70)	0.97
51–60	49 (20.9%)	101 (24.0%)	1.49 (0.30, 7.36)	0.62
>60	1 (0.4%)	3 (0.7%)	2.22 (0.06, 85.50)	0.67
**Education level**				
Doctor	12 (5.1%)	31 (14.5%)	Ref.	
Master	33 (14.0%)	106 (25.2%)	1.82 (0.63, 5.26)	0.27
Bachelor	167 (71.1%)	234 (55.7%)	0.71 (0.25, 2.02)	0.25
Others	23 (9.8%)	49 (11.7%)	1.32 (0.34, 5.18)	0.34
**Professional title**				
Senior	44 (18.7%)	106 (25.2%)	Ref.	
Deputy senior	63 (26.8%)	100 (23.8%)	0.92 (0.45, 1.90)	0.83
Intermediate	84 (35.7%)	125 (29.8%)	1.39 (0.55, 3.48)	0.48
Junior	38 (16.2%)	58 (13.8%)	1.67 (0.53, 5.29)	0.38
Others	6 (2.6%)	31 (7.4%)	5.53 (1.12, 27.41)	**0.04**
**Experience in pharmacy work/year**				
>30	44 (18.7%)	83 (19.8%)	Ref.	
21–30	55 (23.4%)	104 (24.8%)	1.01 (0.39, 2.64)	0.98
11–20	74 (31.5%)	118 (28.1%)	1.34 (0.40, 4.50)	0.63
6–10	44 (18.7%)	78 (18.6%)	0.89 (0.21, 3.69)	0.87
≤ 5	18 (7.7%)	37 (8.8%)	0.51 (0.08, 3.09)	0.64
**Pharmacy administration**				
Favorable	48 (20.4%)	365 (86.9%)	28.10 (17.61, 44.85)	**<0.001**
Unfavorable	187 (79.6%)	55 (13.1%)	Ref.	

The bold values are statistically significant data, mainly for ease of viewing.

a Using multiple logistic regression analysis.

### 3.6 Barriers and suggestions

**Figure 2:**
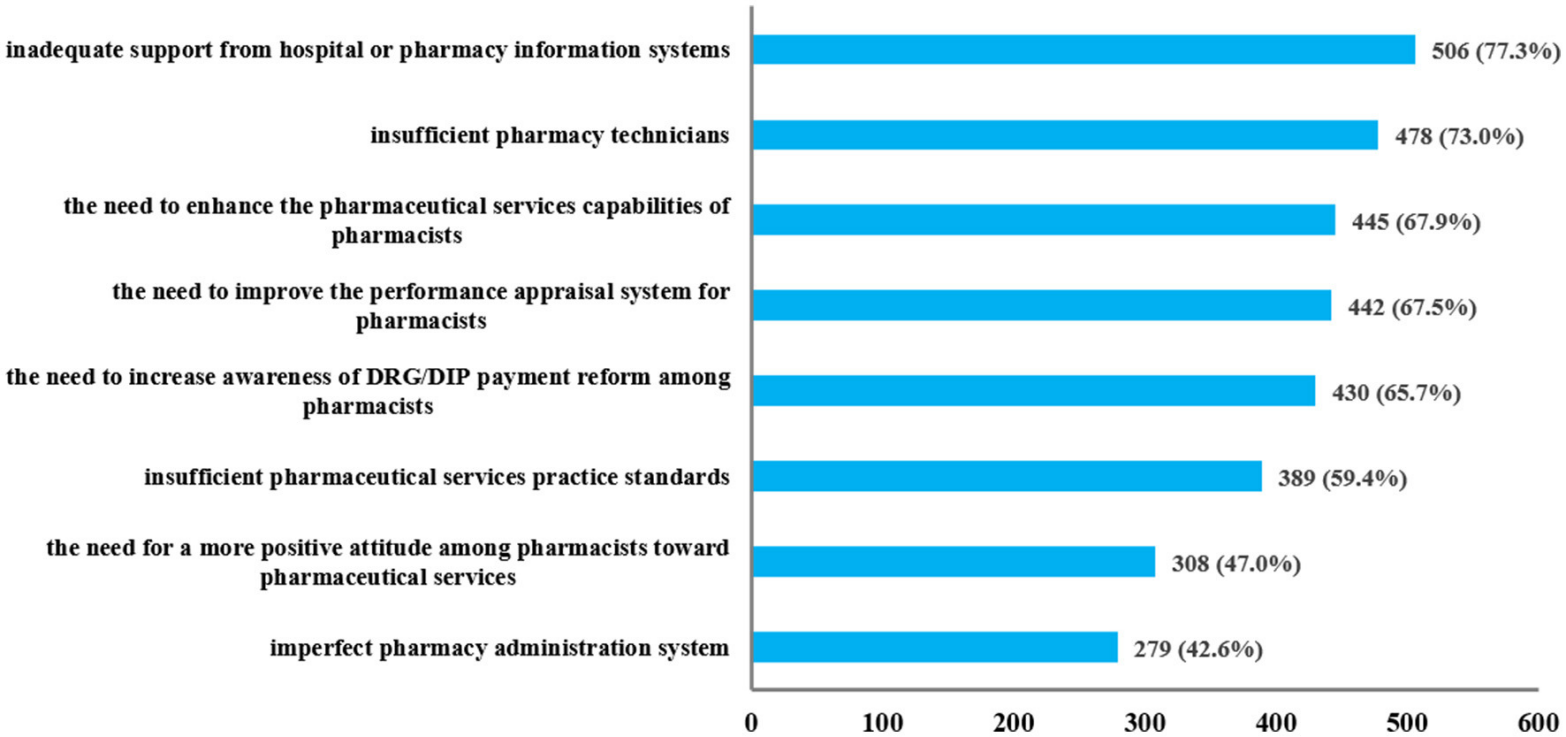
Barriers and challenges existing in pharmacy work under the healthcare payment reform.

Regarding the barriers and challenges existing in pharmacy work under the healthcare payment reform, as shown in Figure 2, most respondents (>70%) pointed out insufficient pharmacy technicians and inadequate support from hospital or pharmacy information systems as major concerns. A significant portion of respondents (>60%, <70%) highlighted the need to enhance the pharmaceutical services capabilities of pharmacists, increase awareness of DRG/DIP payment reform among pharmacists, and improve the performance appraisal system for pharmacists. Additionally, some respondents (>40%, <60%) also expressed concerns about the imperfect pharmacy administration system, the insufficient pharmaceutical services practice standards, and the need for a more positive attitude among pharmacists toward pharmaceutical services.

**Figure 3:**
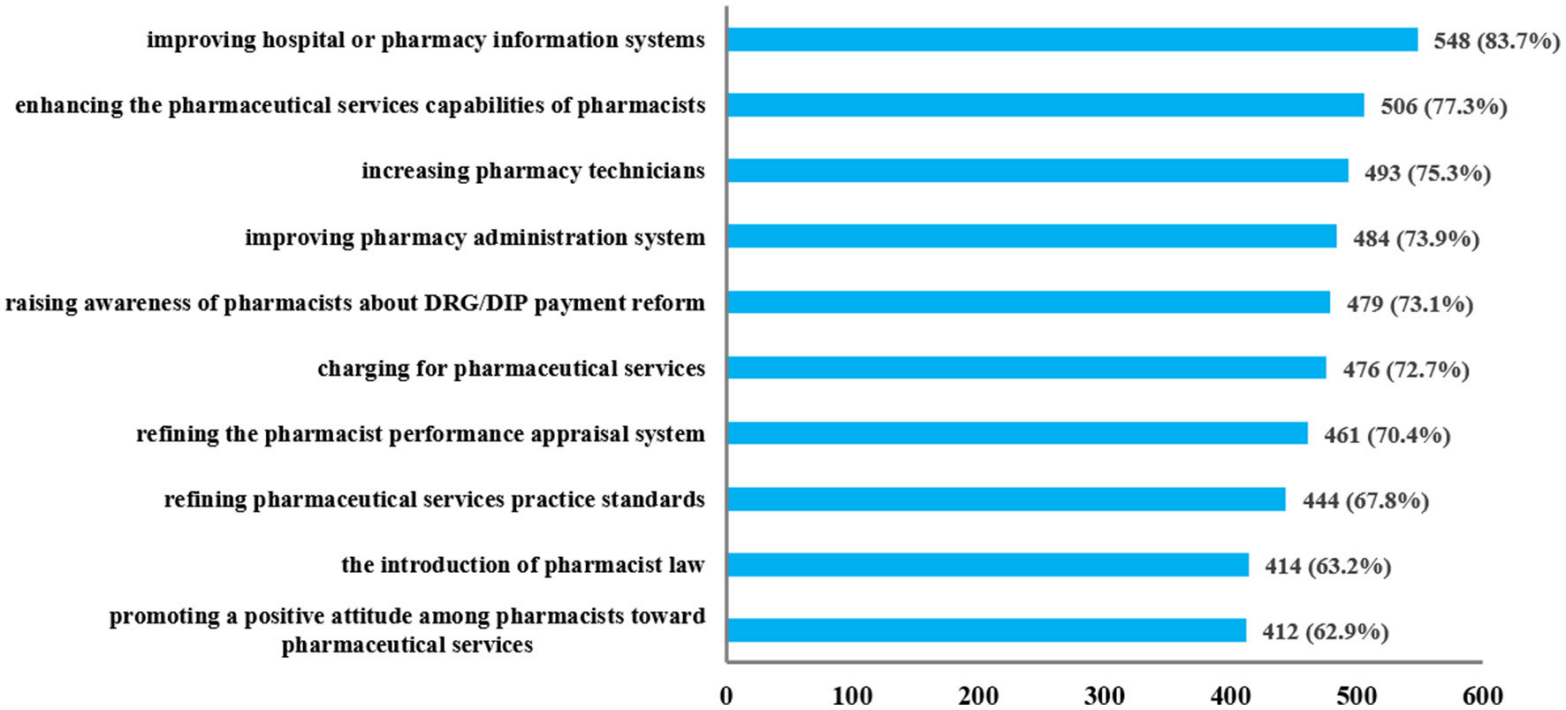
Suggestions to enhance pharmacy work under the healthcare payment reform.

To adapt to the changes brought by healthcare payment reform, respondents provided various suggestions to enhance pharmacy work. As shown in Figure 3, these suggestions focused on information systems, practice standards, pharmacists' awareness, performance appraisal system, and personnel allocation. Most respondents (>70%) proposed increasing pharmacy technicians, improving hospital or pharmacy information systems, enhancing the pharmaceutical services capabilities of pharmacists, raising awareness of pharmacists about DRG/DIP payment reform, charging for pharmaceutical services, refining the pharmacist performance appraisal system, and improving pharmacy administration system. Additionally, many respondents (>60%, <70%) suggested the introduction of pharmacist law, refining pharmaceutical services practice standards, and promoting a positive attitude among pharmacists toward pharmaceutical services.

## 4 Discussion

To the best of the authors' knowledge, this is the first study to investigate the status of hospital pharmacy administration and pharmaceutical services in China under DRG/DIP payment reform. Multiple logistic regression analysis demonstrated that DRG/DIP implemented hospitals were significantly associated with favorable pharmacy administration and pharmaceutical services (*p* < 0.05). For hospitals yet to adopt DRG/DIP payment reform, the potential of pharmacy administration and pharmaceutical services remains to be further explored. Most hospitals have successfully established a foundational framework for pharmacy administration and pharmaceutical services. However, the adoption rate of services required higher levels of digitalization, such as intelligent pharmacy and pharmacy practice in e-hospital, remains relatively low. This study also highlights challenges and provides improvement recommendations at the national, hospital, and pharmacist levels.

To adapt to the evolving landscape of healthcare payment reform, hospitals must undergo a fundamental transformation, with pharmacy administration playing a critical role in this transition. Strengthening pharmacy administration not only promotes rational drug use but also reduces losses resulting from irrational drug use, ultimately improving management efficacy (36, 37). As the healthcare payment reform progresses, pharmacists are playing an increasingly active role in overseeing medical insurance costs. Some studies have shown the positive effects of pharmacist-led pharmaceutical services on patient outcome, such as reducing postoperative pain (38) and hospital stays, as well as alleviating depressive symptoms in women with epilepsy (39). A systematic review of clinical pharmacy services in China showed that these services can reduce medical costs and generate positive economic value (40). Based on these findings, many hospitals anticipate that pharmacists will play a key role in cost management.

Multiple logistic regression analysis revealed that DRG/DIP implemented hospitals exhibited an inclination for favorable pharmacy administration and pharmaceutical services (*p* < 0.05). This trend can be attributed to the higher proportion of tertiary hospitals or hospitals with ≥3,000 beds among the DRG/DIP implemented hospitals. Compared with other hospitals, tertiary hospitals possess greater medical resources, superior conditions for pharmacy administration, heightened awareness of drug safety, and a stronger emphasis on pharmacy administration and pharmaceutical services (15). Moreover, as healthcare payment reform gains traction, hospitals, and medical staff are becoming increasingly cost-conscious (41). This drives hospital pharmacy departments to leverage the DRG/DIP payment system as a foundation for strategically reducing medical costs while ensuring treatment safety and efficacy. For hospitals yet to adopt the DRG/DIP payment reform, the full potential of pharmacy departments in pharmacy administration and pharmaceutical services remains untapped, necessitating further enhancement. Additionally, since DRG/DIP were introduced in 2019 and 2020, some hospitals have been implementing DRG/DIP for a long time. Further research is needed to establish a causal relationship between DRG/DIP implementation and pharmacist involvement.

In recent years, with the increasing demand for rational drug use and deepening of health system reform, hospitals pharmaceutical services in China has undergone rapid evolution. Guided by policies and regulations, the overall hospital pharmaceutical services has reached a certain level, expanding in scope and depth (42). Our study found that most hospitals have successfully established basic pharmacy administration and pharmaceutical services frameworks. However, certain institutions still require improvements in these areas. Notably, less than 50% of hospitals offer medication therapy management, reflecting a shortage of clinical pharmacists (20, 43). Only 30% of hospitals provide intelligent pharmacy and pharmacy practice in e-hospital, while fewer than 20% offer hospital medication home delivery service. These services require higher levels of digitalization (44), posing challenges for some hospitals. Furthermore, less than 40% of hospitals provide therapeutic drug monitoring, and less than 30% conduct pharmacogenetic testing. These services require specialized facilities and trained operators, making implementation challenging for primary hospitals (45). Additionally, despite their potential benefits, these services are in low demand in primary hospitals, resulting in limited clinical applications (46). DRG/DIP implemented hospitals tend to provide a broader range of pharmacy administration and pharmaceutical services compared to non-DRG/DIP implemented hospitals, consistent with previous findings. Among DRG/DIP implemented hospitals, a higher proportion of tertiary hospitals can provide a wider range of pharmaceutical services (47).

China's healthcare payment reform is progressing actively. Among the 655 hospitals surveyed, 73.4% (481) have implemented DRG/DIP payment reform, while 26.6% (174) have not. Compared with non-DRG/DIP implemented hospitals, most of the DRG/DIP implemented hospitals are tertiary hospitals or with ≥3,000 beds. This suggests that hospitals with higher grade and more extensive facilities are more proactive in responding to the healthcare payment reform, possibly related to the inclusion of DRG-related indicators in tertiary hospitals accreditation standards (48). According to economic division, eastern and central China exhibit a higher proportion of DRG/DIP implemented hospitals, which is related to economic development to a certain extent. Conversely, western China, characterized by relatively lower level of economic development (20), possesses fewer DRG/DIP implemented hospitals.

Among the 655 respondents, 27.8% (182/655) held a master's or doctoral degree, with a higher proportion observed in DRG/DIP implemented hospitals (32.4%, 156/481) and a lower proportion in non-DRG/DIP implemented hospitals (14.9%, 26/174). Currently, the hospital pharmacy personnel in China still mainly have a bachelor's degree (15). Pharmacy courses in Chinese universities often follow traditional training model with chemistry teaching as the core, leading to the lack of clinical reasoning, communication, and practice skills in pharmacy students (20). This challenge is also reflected in the survey, with 77.3% of respondents stating the necessity of improving pharmaceutical services capabilities of pharmacists. Encouraging and supporting collaborations between hospitals and universities is essential to the development of targeted, forward-thinking training programs and the cultivation of high-level pharmacy professionals.

In this study, respondents from various hospitals raised barriers and suggestions for enhancing hospital pharmacy work. The nature of these challenges and proposed solutions were discussed from the level of the nation, hospitals, and pharmacists.

At the national level, over 40% of respondents identified the imperfect pharmacy administration system, the insufficient pharmaceutical services practice standards as challenges in pharmacy work. The introduction of pharmacist law holds significance for clarifying the rights and obligations of pharmacists, rationalizing the management system, and promoting the high-quality development of pharmaceutical services (34). And the rational charging for pharmaceutical services is conducive to compensating essential costs, increasing departmental revenue, and fully motivating pharmacists' work enthusiasm and subjective initiative (25, 34). Research on the status of clinical pharmacy services in tertiary hospitals in China indicates the needs for further improvements in the construction of China's clinical pharmacy services in terms of system, hardware, and personnel (49). In recent years, a series of policies detailing pharmacy administration and pharmaceutical services practice standards were issued. In 2021, five kinds of pharmaceutical services practice standards were formulated. In 2023, the formulation of pharmacist law was included in the plan of the Standing Committee of the 14th National People's Congress. In the same year, three kinds of pharmaceutical services were included in charging items at the national level for the first time. Subsequently, Fujian and Hubei provinces launched trial implementation of pharmaceutical services charging. These policies are crucial for promoting the development of pharmacy administration and pharmaceutical services.

At the level of hospitals, over 60% of respondents perceived issues such as insufficient pharmacy technicians, imperfect performance appraisal system for pharmacists, and inadequate support from hospital or pharmacy information systems. Increasing pharmacy technicians, especially high-level talents, can meet the demands of pharmaceutical services and deliver refined and comprehensive services. Establishing a pharmacist performance appraisal system tailored to clinical needs and the characteristics of pharmaceutical services, can stimulate the enthusiasm of pharmacists for patient care and clinical service (15). In the context of the healthcare payment reform, the development of information technology is crucial for constructing the DRG/DIP payment system and the new mode of rational drug use management. Improving hospital or pharmacy information systems to achieve integration, standardization and process of pharmacy administration is pivotal for delivering intelligent, accurate and mobile pharmaceutical services.

At the level of pharmacists, over 40% of respondents felt that pharmacists should exhibit a more positive attitude toward pharmaceutical services, while over 60% believed that pharmaceutical services capabilities of pharmacists needed enhancing and awareness of pharmacists about DRG/DIP payment reform needed raising. In a qualitative interview study, approximately half of the surveyed clinical pharmacists exhibited insufficient familiarity with DRG/DIP payment principles (37). The imperfect performance appraisal system for pharmacists may lead to the lack of positive attitude. Addressing these issues at both the national and hospital levels will contribute to boosting pharmacists' work enthusiasm. Improving the pharmaceutical services capabilities of pharmacists requires not only individual efforts but also external support, such as optimizing the training mode of pharmacy students, enhancing the continuing education of clinical pharmacists, reforming the training program of clinical pharmacists, and consistently conducting training in pharmaceutical services.

There are still some limitations in this study. Firstly, as this is a cross-sectional study, in which exposure and outcome are measured simultaneously, it is not possible to establish causal relationships between the analyzed variables. This limitation should be considered when interpreting the findings. Secondly, it is essential to note the inherent limitations of sampling methods. Using convenience sampling may lead to selection bias, potentially overrepresenting hospitals with readily available pharmacy leaders. The pharmacy professionals who complete the questionnaire were designated by the pharmacy department heads, rather than selected according to certain principles. This may also limit representativeness. Further research is needed to confirm the conclusions. Thirdly, the questionnaire fails to provide adequate explanations for key items, which may result in varied interpretations among respondents and consequently lead to understanding question-reading deviations. Fourthly, the implementation effects of hospital pharmacy administration and pharmaceutical services relied on the self-assessment of the respondents. Although anonymous method was employed in the investigation process, and the personal characteristics of respondents were included to explore predictors of favorable results, there may still be a deviation of social expectations. Additionally, the study did not explore potential tensions between cost-containment and optimal pharmaceutical services. High-quality clinical evidence on the effectiveness of pharmacy administration/pharmaceutical services under DRG/DIP payment reform is needed. Despite these limitations, the findings still provide valuable insights.

## 5 Conclusions

The ongoing healthcare payment reform in China is in the process of active promotion. According to the findings of this study, the implementation of DRG/DIP payment reform positively correlates with elevated levels of hospital pharmacy administration and pharmaceutical services. Furthermore, the characteristics of hospitals exert a more substantial influence on achieving high-quality pharmacy administration and pharmaceutical services than the personal characteristics of pharmacists. To effectively adapt the healthcare payment reform, it is suggested that the pharmacy department should strategically leverage DRG/DIP as a starting point. This involves continuous enhancement of pharmaceutical services capabilities, fostering the advancement of pharmacy administration, and realizing value throughout the ongoing healthcare payment reform.

## Data Availability

The raw data supporting the conclusions of this article will be made available by the authors, without undue reservation.
