# Supplementary material for: Effects of DRG/DIP payment reform on hospital pharmacy administration and pharmaceutical services in China: a multicenter cross-sectional study
Source: Front Public Health. 2025 Jul 11;13:1585279. doi: 10.3389/fpubh.2025.1585279 (PMC12289633; doi:10.3389/fpubh.2025.1585279)
Supplement: Supplementary file 1 [file Data_Sheet_1.docx]

Supplementary File 1

# Questionnaire used in the study (Chinese version)

**一、个人信息**

1. 您的性别：

□男 □女

1. 您的年龄（岁）：

□≤30 □31-40 □41-50 □51-60 □>60

1. 您的学历：

□博士 □硕士 □学士 □其他

1. 您的职称：

□高级 □副高级 □中级 □初级 □其他

1. 您的药学工作年限：

□>30 □21-30 □11-20 □6-10 □≤5

**二、医疗机构信息**

1. 医疗机构等级：

□三级 □非三级

1. 医疗机构类别：

□综合医院 □专科医院

1. 医疗机构床位数：

□≥3000 □<3000

1. 医疗机构所在城市地理区域：

□东部 □中部 □西部

**三、医疗机构药事管理与药学服务现状**

1. 医疗机构的医保支付方式：（可多选）

□按疾病诊断相关分组付费（DRG） □按项目付费

□按病种分值付费（DIP） □总额预付

□其他_________

1. 医疗机构药学部门在DRG/DIP支付管理体系中的具体职责：（可多选，仅适用于上一个问题中选择 DRG 或 DIP 的填写人）

□与临床科室沟通 □医院处方集管理

□临床路径制定 □用药监测

□药物评价指标设定 □其他_________

□无明确职责

1. 医疗机构药学部门提供的药事管理：（可多选）

□超说明书用药管理 □医院处方集管理

□药品不良事件管理 □抗菌药物管理

□向医生解释药物政策 □临床路径制定

□药物临床综合评价 □处方点评

□首选药物方案的制定 □其他_________

1. 医疗机构药学部门提供的药学服务：（可多选）

□单剂量摆药 □用药咨询

□静脉用药集中调配 □处方审核

□药学查房 □药学会诊

□多学科诊疗 □药学门诊

□药物治疗管理 □互联网药学服务

□治疗药物监测 □智慧药房

□医院药品配送到家 □药物基因检测

□对临床医护或患者提供合理用药讲课

□其他_________

（请根据您的同意程度选择相应的选项）

1. 您认为您所在的医疗机构具备完善的药事管理制度？

□非常同意 □同意 □中立 □不同意 □非常不同意

1. 您认为您所在的医疗机构具备完善的药学服务规范？

□非常同意 □同意 □中立 □不同意 □非常不同意

1. 您认为您所在的医疗机构药学部门积极参与DRG/DIP支付方式改革？

□非常同意 □同意 □中立 □不同意 □非常不同意

1. 您认为您所在医疗机构的药学服务满足了患者治疗需求？

□非常同意 □同意 □中立 □不同意 □非常不同意

1. 您认为您所在医疗机构的药学服务降低了患者药品费用？

□非常同意 □同意 □中立 □不同意 □非常不同意

1. 您认为您所在医疗机构的药学服务促进了合理用药？

□非常同意 □同意 □中立 □不同意 □非常不同意

**四、挑战与建议**

1. 医保支付方式改革背景下药学工作存在的问题与挑战：（可多选）

□药事管理制度不健全

□药学服务技术规范不完善

□医院或药学信息系统支持不足

□药师对药学服务的态度需要更积极

□药师的药学服务能力需要提升

□药师对DRG/DIP支付方式改革的认知需要加强

□药师绩效考核体系需要完善

□药学技术人员不足

□其他_________

1. 医保支付方式改革背景下对加强药学工作的建议：（可多选）

□完善药师管理制度

□完善药学服务技术规范

□完善医院或药房信息系统

□提升药师对药学服务的积极态度

□提升药师药学服务能力

□提高药师对DRG/DIP支付方式改革的认知

□完善药师绩效考核体系

□增加药学技术人员

□药学服务收费

□出台药师法

□其他_________

# Questionnaire used in the study (English version)

The Chinese version of the questionnaire was translated into English version by a professional native speaker with expertise in the field. The English terminology for specialized concepts, such as pharmacy administration and pharmaceutical services, was derived from official documents and group standards issued by authoritative bodies, including the National Health Commission and the Chinese Hospital Association. The Chinese Hospital Association, a national, industry-based social organization under the National Health Commission, developed a comprehensive group standard system for "Pharmacy Administration and Pharmaceutical Services in Medical Institutions", comprising 45 group standards. For a clearer understanding, relevant descriptions of these terms have been added to the end of the questionnaire in questionnaire instructions section.

**Section I. Personal Information**

1. Gender:

□Male □Female

1. Age (years)：

□≤30 □31-40 □41-50 □51-60 □>60

1. Education level:

□Doctor □Master □Bachelor □Others

1. Professional title:

□Senior □Deputy senior □Intermediate □Junior □Others

1. The length of experience in pharmacy work: ___year(s)

□>30 □21-30 □11-20 □6-10 □≤5

**Section II. Medical Institution Information**

1. Hospital grade:

□Tertiary hospital □Non-tertiary hospital

1. Hospital category:

□General hospital □Specialized hospital

1. Number of hospital beds:

□≥3000 □<3000

1. Region of the city where the hospital is located:

□East □Middle □West

**Section III. Current Status of Pharmacy Administration and Pharmaceutical Services in Medical Institutions**

1. The medical insurance payment methods of hospital: (Multiple choice)

□Diagnosis Related Groups (DRG) □Fee for Service

□Diagnosis-Intervention Packet (DIP) □Global Budget

□Others_________

1. The roles of hospital pharmacy departments in the DRG/DIP payment management system: (Multiple choice, only for respondents who choose DRG or DIP in the previous question)

□Communication with clinical departments □Hospital formulary management

□Clinical pathway development □Medication monitoring

□Medication evaluation index setting □Others_________

□No clear responsibilities

1. Pharmacy administration provided by the hospital pharmacy departments: (Multiple choice)

□Off-label medication management □Hospital formulary management

□Adverse drug event management □Antimicrobial stewardship

□Interpreting drug policy for physicians □Clinical pathway development

□Comprehensive medicine-use evaluation □Prescription evaluation

□Preferred pharmacologic regimen development □Others_________

1. Pharmaceutical services provided by the hospital pharmacy departments: (Multiple choice)

□Unit dose dispensing system □Medication consultant

□Pharmacy intravenous admixture □Prescription review

□Pharmaceutical ward round □Pharmaceutical consults

□Multi-disciplinary treatment □Pharmaceutical clinic

□Medication therapy management □Pharmacy practice in e-hospital

□Therapeutic drug monitoring □Intelligent pharmacy

□Hospital medication home delivery service □Pharmacogenetic testing

□Providing lectures on rational drug use for medical staff or patients

□Others_________

(Please check the box that corresponds with your agreement.)

1. Do you think your hospital have a sound pharmacy management system?

□Strongly agree □Agree □Neutral □Disagree □Strongly disagree

1. Do you think your hospital have complete pharmaceutical care practice standards?

□Strongly agree □Agree □Neutral □Disagree □Strongly disagree

1. Do you think your hospital have active involvement of pharmacy department under DRG/DIP payment reform?

□Strongly agree □Agree □Neutral □Disagree □Strongly disagree

1. Do you think the hospital pharmaceutical services meet the patient treatment?

□Strongly agree □Agree □Neutral □Disagree □Strongly disagree

1. Do you think the hospital pharmaceutical services reduce drug costs for patients?

□Strongly agree □Agree □Neutral □Disagree □Strongly disagree

1. Do you think the hospital pharmaceutical services promote rational drug use?

□Strongly agree □Agree □Neutral □Disagree □Strongly disagree

**Section IV. Challenges and Recommendations**

1. Problems and challenges existing in pharmacy work under the healthcare payment reform: (Multiple choice)

□Imperfect pharmacy management system

□Insufficient pharmaceutical care practice standards

□Inadequate support from hospital or pharmacy information systems

□The need for a more positive attitude among pharmacists toward pharmaceutical care

□The need to enhance the pharmaceutical care capabilities of pharmacists

□The need to increase awareness of DRG/DIP payment reform among pharmacists

□The need to improve the performance appraisal system for pharmacists

□Insufficient pharmacy technicians

□Others_________

1. Recommendations to enhance pharmacy work under the healthcare payment reform: (Multiple choice)

□Improving pharmacy management system

□Refining pharmaceutical care practice standards

□Improving hospital or pharmacy information systems

□Promoting a positive attitude among pharmacists toward pharmaceutical care

□Enhancing the pharmaceutical care capabilities of pharmacists

□Raising awareness of pharmacists about DRG/DIP payment reform

□Refining the pharmacist performance appraisal system

□Increasing pharmacy technicians

□Charging for pharmaceutical care

□The introduction of pharmacist law

□Others_________

**Questionnaire Instructions**

1. Professional title ^[1]^: senior (chief pharmacist), deputy senior (associate chief pharmacist), intermediate (pharmacist-in-charge), junior (pharmacist and assistant pharmacist), others (respondents not attain a professional title yet).

2. Off-label medication management: The oversight and control of the use of pharmaceuticals for unapproved indications or in an unapproved age group, dosage, or route of administration.

3. Hospital formulary management: Offering expert pharmaceutical assessments and recommendations to hospital formulary.

4. Adverse drug event management: The systematic process of identifying, evaluating, monitoring, and preventing adverse drug events to ensure patient safety and optimize therapeutic outcomes.

5. Antimicrobial stewardship: A coordinated program that promotes the appropriate use of antimicrobials (including antibiotics), improves patient outcomes, reduces microbial resistance, and decreases the spread of infections caused by multidrug-resistant organisms.

6. Interpreting drug policy for physicians: Presentations delivered to physicians, nurses, and other healthcare providers regarding drug policies.

7. Clinical pathway development: Involvement in the design and establishment of standardized care plans for patients with specific clinical problems.

8. Comprehensive medicine-use evaluation ^[2]^: A multidimensional and multi-level evidence-based assessment where the evaluating subject selects appropriate theoretical frameworks, methodologies, and tools to collect and analyze data and information related to drug utilization and supply within medical institutions, thereby appraising clinical efficacy and the actual implementation effectiveness of drug policies.

9. Prescription Evaluation ^[2]^: A systematic process involving the assessment of the standardization of prescription writing and the appropriateness of clinical drug use (including indications, drug selection, administration route, dosage, drug interactions, incompatibility, etc.) according to relevant regulations and technical standards. This process aims to identify existing or potential problems, and to develop and implement interventions and improvement measures to promote rational clinical drug application.

10. Preferred pharmacologic regimen development: The formulation of preferred therapeutic strategies for specific disease categories classified under Diagnosis-Related Group (DRG) or Diagnosis-Intervention Packet (DIP) payment systems.

11. Unit dose dispensing system ^[3]^: A system in inpatient drug therapy where all medications taken by a patient are dispensed by pharmacy personnel in single-unit packages. The dispensing staff repackage various solid dosage forms prescribed to patients into single administration doses, often with the help of an automatic medicine packing machine.

12. Medication consultant ^[2]^: The process whereby pharmacists utilize their professional pharmaceutical knowledge and tools to provide drug information to patients, their families, and healthcare professionals, disseminate knowledge on rational drug use, and communicate on medication-related issues.

13. Pharmacy intravenous admixture ^[2]^: A process within the pharmacy department of a medical institution where, following physician prescriptions or medication orders and after pharmacist review and intervention for appropriateness, pharmacy professionals prepare intravenous medications by admixing them in a clean environment under aseptic conditions, resulting in ready-to-use infusions for direct clinical intravenous administration.

14. Prescription review ^[2]^: A pharmaceutical technical service wherein pharmacy professionals, leveraging their specialized knowledge and practical skills, conduct a review of prescriptions issued by physicians during diagnostic and therapeutic activities for legality, standardization, and appropriateness, in accordance with relevant laws, regulations, and technical specifications, before making decision on whether to dispense the medication.

15. Pharmaceutical ward round ^[2]^: The process of clinical pharmacists conducting patient rounds within hospital wards with the purpose of promoting rational drug use.

16. Pharmaceutical consults ^[2]^: A pharmaceutical service where pharmacists, upon invitation from clinical departments or medical affairs units, provide pharmaceutical services such as optimizing patients' drug therapy regimens and drug monitoring as required for diagnosis and treatment.

17. Multi-disciplinary treatment ^[2]^: A collaborative diagnostic and treatment work model where a team of experts from multiple specialties convenes regularly. Through meetings, they comprehensively consider the patient's specific situation to formulate an individualized treatment plan, which is then executed by a single department or jointly by several departments.

18. Pharmaceutical clinic ^[2]^: A specialized outpatient service where hospital pharmacists provide patients with a range of professional pharmaceutical services, including medication assessment, medication consultant, patient education, and recommendations for medication regimen adjustments.

19. Medication therapy management ^[2]^: Pharmacy personnel provide patients with a series of professional services such as medication education, consultation and guidance, thereby enhancing medication adherence, preventing medication errors, and assisting patients in self-medication management to achieve therapeutic goals and ensure medication safety.

20. Pharmacy practice in e-hospital ^[2]^: A process involving pharmacy professionals in medical institutions who utilize their specialized knowledge and practical skills, in accordance with relevant regulations and technical standards, to review electronic prescriptions issued by physicians during internet-diagnosis and treatment, and perform a series of pharmaceutical services such as prescription dispensing, medication verification and issuance, patient education, medication consultant, identification and management of adverse drug reactions, and chronic disease medication therapy management.

21. Therapeutic drug monitoring ^[2]^: The practice of measuring drug concentrations, pharmacological markers, or pharmacodynamic indicators in a patient's system and utilizing quantitative pharmacological models and taking the drug therapeutic window as the benchmark, to develop individualized dosing regimens. Its core is personalized drug therapy.

22. Intelligent pharmacy ^[4]^: A pharmacy model that applies information technologies such as big data, cloud computing, and the Internet of Things, utilizing various data resources to support scientific decision-making. It incorporates intelligent control systems for traceable and refined management of pharmaceutical processes including requisition, storage, dispensing, verification, distribution, and administration, offering patients pharmaceutical services like prescription review, medication dispensing, medication reconciliation, medication instructions, medication reminders, and medication follow-up, thereby achieving intelligent management of work tasks and environmental conditions.

23. Hospital medication home delivery service ^[5]^: A service model where medical institutions collaborate with legally qualified courier and logistics companies to deliver prescribed medications directly to a patient's designated address (e.g., residence, workplace). This involves processes such as medication picking, checking, packaging, sealing, dispatching, and transportation to deliver medications to the consumer's specified location for signed receipt, based on their medication purchase needs.

24. Pharmacogenetic testing ^[6]^: A detection technique employing specific molecular biology methods to examine genes related to drug metabolism enzymes and drug targets in the human body. This testing predicts the patient's responsiveness to drugs (including efficacy and risk of adverse reactions), thereby assisting clinicians in formulating individualized medication plans concerning drug selection, dosage adjustment, and combination therapy.

25. Providing lectures on rational drug use for medical staff or patients: The process by which pharmacists provide patients or medical staff with guidance on rational drug use and disseminate knowledge about appropriate medication practices to enhance medication adherence, reduce the incidence of medication errors, and ensure medical quality and safety.

**References:**

[1] Ministry of Human Resources and Social Security of China. Guiding opinions on deepening the reform of the health professional technical position system. Accessed 30 July 2022. https://www.mohrss.gov.cn/wap/zc/zcwj/202108/t20210804_420042.html

[2] Chinese Hospital Association. Notice on the release of the fourth batch of 7 group standards on " Pharmacy Management and Pharmaceutical Services in Medical Institutions". Accessed 30 July 2022. https://www.cha.org.cn/site/content/f964553759710b2147552d5a49064d4e.html

[3] Qiu T, Yan Y, Zhang Y, Zhang C, Cheng Y, Kong X, et al. Construction and optimization of automatic checking mode for unit dose dispensing system of oral drugs. China Pharmacy. 2023;34:2018-21. doi: 10.6039/j.issn.1001-0408.2023.16.19

[4] Anhui Provincial Administration for Market Regulation. Guideline for the construction of intelligent pharmacy. Accessed 30 July 2022. https://std.samr.gov.cn/db/search/stdDBDetailed?id=BF4960B9661558D3E05397BE0A0A996E

[5] National Medical Products Administration of China. Announcement on the release of appendix 6 of the good supplying practice for pharmaceuticals: quality management of pharmaceutical retail distribution. Accessed 30 July 2022. https://www.nmpa.gov.cn/yaopin/ypggtg/20221130200516116.html

[6] Anhui Provincial Administration for Market Regulation. Specification for the individualized pharmaceutical care guided bypharmacogenomics testing of medical institution. Accessed 30 July 2022. https://std.samr.gov.cn/db/search/stdDBDetailed?id=1A443F40A7CC52F2E06397BE0A0ADE89
