# Supplementary material for: Effects of DRG/DIP payment reform on hospital pharmacy administration and pharmaceutical services in China: a multicenter cross-sectional study
Source: Front Public Health. 2025 Jul 11;13:1585279. doi: 10.3389/fpubh.2025.1585279 (PMC12289633; doi:10.3389/fpubh.2025.1585279)
Supplement: Supplementary file 2 [file Table_1.docx]

Supplementary File 2**Table S1 The assessment of implementation effects of hospital pharmacy administration and pharmaceutical services (N=655)**

| **Item** | **Strongly agree**  **n (%)** | **Agree**  **n (%)** | **Neutral**  **n (%)** | **Disagree**  **n (%)** | **Strongly disagree n (%)** |
| --- | --- | --- | --- | --- | --- |
| Do you think your hospital have a sound pharmacy management system? | 188 (28.7) | 338 (51.6) | 110 (16.8) | 13 (2.0) | 6 (0.9) |
| Do you think your hospital have complete pharmaceutical care practice standards? | 129 (19.7) | 326 (49.8) | 163 (24.9) | 32 (4.9) | 5 (0.8) |
| Do you think your hospital have active involvement of pharmacy department under DRG/DIP payment reform? | 135 (20.6) | 334 (51.0) | 158 (24.1) | 25 (3.8) | 3 (0.5) |
| Do you think the hospital pharmaceutical services meet the patient treatment? | 97 (14.8) | 336 (51.3) | 178 (27.2) | 39 (6.0) | 5 (0.8) |
| Do you think the hospital pharmaceutical services reduce drug costs for patients? | 151 (23.1) | 364 (55.6) | 122 (18.6) | 15 (2.3) | 3 (0.5) |
| Do you think the hospital pharmaceutical services promote rational drug use? | 201 (30.7) | 385 (58.8) | 59 (9.0) | 9 (1.4) | 1 (0.2) |
